# Molecular Characterization of Oxaliplatin‐Induced Peripheral Neurotoxicity: The Complex Spectrum of Painful Manifestations

**DOI:** 10.1111/jns.70078

**Published:** 2025-11-15

**Authors:** Eleonora Pozzi, Maria Pina Serra, Marianna Boi, Annalisa Canta, Alessia Chiorazzi, Chiara Capelli, Chiara Invernizzi, Elisa Ballarini, Virginia Rodriguez‐Menendez, Margherita Francesca Kraus, Marina Quartu, Guido Cavaletti, Paola Alberti

**Affiliations:** ^1^ Experimental Neurology Unit, School of Medicine and Surgery Monza Italy; ^2^ NeuroMI (Milan Center for Neuroscience) Milan Italy; ^3^ Section of Cytomorphology, Department of Biomedical Sciences, University of Cagliari Italy; ^4^ Istituto di Scienze e Tecnologie Chimiche “Giulio Natta” SCITEC CNR Milan Italy; ^5^ PhD Program in Neuroscience, University of Milano‐Bicocca Monza Italy

**Keywords:** hyperexcitability, immunolabelling, nerve excitability testing, neuropathic pain, oxaliplatin neuropathy, oxaliplatin neurotoxicity, TRPV1

## Abstract

**Background and Aims:**

Oxaliplatin (OHP) induced peripheral neurotoxicity (OIPN) is a complex spectrum comprising an acute and a chronic form. Acute OIPN leads to unpleasant transient sensations in the 24–72 h after chemotherapy, due to a temporary dysfunction in ion channels (i.e., axonal hyperexcitability in the absence of anatomical damage). Whereas chronic OIPN is characterized by painful manifestations. Literature data showed that a more pronounced acute OIPN could be an early predictor of chronic OIPN; thus, acute OIPN is becoming a possible target to prevent chronic OIPN. We went back to the bench side to characterize the complexity of painful phenomena experienced by OHP‐treated patients.

**Methods:**

Female OHP‐treated (3 mg/Kg, 2qwx4ws, iv) and control rats (*n* = 10/group) were studied. Acute OIPN was detected via nerve excitability testing (NET), whereas chronic OIPN was assessed via behavioral tests, nerve conduction studies (NCS), and neuropathology (including immunohistochemistry on lumbar spinal cord specimens) both at the end of the full chemotherapy treatment (4 weeks) and at 6 weeks of follow‐up. NET was also performed 1 week after treatment completion.

**Results:**

NET alterations related to acute OIPN were resolved within a week after chemotherapy. Whereas, chronic OIPN encountered only partial recovery over time, with prominent small fiber damage. Immunolabeling of the spinal cord at the end of treatment and after the follow‐up period was consistent with persistent neuropathic pain.

**Interpretation:**

Our data supports the statement that unpleasant manifestations due to acute and chronic OIPN mirror different underlying phenomena and assessment as two separate entities should be considered in both clinical and preclinical studies.

## 1 Introduction

Oxaliplatin (OHP) is one of the cornerstone drugs to treat colorectal cancer and is therefore one of the most widely prescribed agents in Oncology daily practice. However, its clinical use is limited by neurological sequelae that specifically affect the peripheral nervous system: OHP‐induced peripheral neurotoxicity (OIPN) has a rather peculiar clinical phenotype, consisting of both a transient hyperexcitability syndrome (“acute” OIPN) and a chronic syndrome characterized by persistent axonal damage [1, 2, 3]. The persistence of chronic OIPN was demonstrated by a long‐term follow‐up study by Briani et al. [4]: relying on robust outcome measures they observed that a consistent proportion of the study population showed OIPN signs/symptoms 2 years after chemotherapy discontinuation.

Chronic OIPN is part of the broader entity referred to as chemotherapy‐induced peripheral neurotoxicity (CIPN), which is related to a large variety of anticancer drugs (platinum drugs, taxanes, vinca alkaloids, proteasome inhibitors and thalidomide) [5]. While the clinical manifestations may vary according to the drug administered, CIPN is typically characterized by common key features of a length‐dependent sensory polyneuropathy/neuronopathy, with little to no motor involvement [2]. Depending on the extent of damage to small or large myelinated fibers patients can develop sensory loss/abnormalities for different sensory modalities. Small fiber involvement is often associated with neuropathic pain, whereas large fiber damage primarily leads to altered proprioception resulting in impairment in manual dexterity and gait/balance, but without reduced strength [6, 7, 8].

In contrast, acute OIPN is a phenomenon unique to OHP. As soon as the first chemotherapy cycle, patients very frequently experience transient symptoms such as cold‐induced paresthesia, cramps, fasciculations, jaw spasm, which typically resolve between cycles, usually in the 24–72 h after each iv administration [9, 10, 11]. These manifestations can be related to a state of transient axonal hyperexcitability caused by ion channel dysfunctions [1, 12]. Evidence from small clinical cohorts using nerve excitability testing (NET), a technique that enables to assess indirectly axonal properties and ion channel dysfunctions, has revealed a pattern consistent with functional and reversible alterations in sodium‐voltage operated ion channels [13, 14, 15]. Similar findings have been reported in rodent models [16, 17]. Moreover, animal studies have also demonstrated that interventions targeting acute OIPN—a phenomenon distinct from both axonal damage and neuropathic pain—can prevent the development of chronic OIPN [18]. It has been suggested, in fact, that axonal hyperexcitability due to acute OIPN leads to the activation of the sodium calcium‐exchanger2 (NCX2) *reverse mode*: in the case of altered excitability, as induced by OHP, NCX2 extrudes sodium from cells and exchanges it with calcium (in the *forward mode*, instead, it extrudes calcium in exchange for sodium intake), leading to a potentially calcium‐related neurotoxicity [19]. In line with this, preliminary in vitro data were published showing a promising neuroprotection against OIPN, inhibiting NCX2 via SEA0400 [18, 19].

The aim of our study was, therefore, to provide a rigorous preclinical morphological and functional characterization of all painful manifestations related to the full OIPN spectrum.

## 2 Materials and Methods

### 2.1 Animal Housing and Treatment

A total of 20 female Wistar rats (Envigo, Udine, Italy) of 175–200 g at arrival were caged in a temperature‐ and relative humidity‐controlled room (22°C ± 2°C and 55% ± 10%, respectively) with a 12 h light/dark cycle and were allowed access to food and water *ad libitum*. Animal care and treatment met the guidelines set by the Italian Ministry of Health (D. L.vo 26/2014, *Gazzetta Ufficiale della Repubblica Italiana*, n.61, 14 March 2014) and the international laws and policies (European Union directive 2010/63/UE; Guide for the Care and Use of Laboratory Animals, U.S. National Research Council, 1996). All experimental procedures were also authorized by the Italian Ministry of Health (authorization number N. 920/2016‐PR, 5 October 2016).

After 1 week quarantine period, rats were divided into two groups: control group (naïve, CTRL, *n* = 10) and oxaliplatin‐treated group (OHP, *n* = 10).

Oxaliplatin powder (OHP, Debiopharm, Lausanne, Switzerland) was dissolved in a 5% glucose solution to obtain a concentration of 3 mg/Kg and was intravenously administered in the tail vein twice a week for 4 weeks (2qwx4ws) at a volume of 1 mL/Kg.

Body weight was measured before each drug injection for a general health check and dose adjustment. All the rats were examined daily also during the 6‐week follow‐up period.

At the end of the treatment and follow‐up period, rats were euthanized by overdose of carbon dioxide (CO_2_) inhalation, and tissue samples (caudal nerves, spinal cord, L4–L5 lumbar dorsal root ganglia (DRG), skin biopsies) were collected.

### 2.2 Study Design

The outcome measure to detect and characterize OIPN hyperexcitability syndrome was NET, whereas chronic OIPN was assessed via nerve conduction studies (NCS), behavioural tests for mechanical allodynia, and morphological and morphometrical analyses on caudal nerves, DRG, and spinal cord specimens.

*At baseline*, to ensure homogeneity between the 2 groups, NCS were performed. *After the 4‐week treatment period* as well as *after 6 weeks of follow‐up* following treatment completion, data were collected as mentioned above to fully characterize/detect both acute and chronic OIPN.

Sample size for each group was calculated on the basis of nerve conduction velocity (NCV) reference values of our laboratory [20], assuming that the relevant difference between CTRL and OHP groups is 5 m/s (standard deviation = 7); thus, if a 2‐sided 5% alpha and an 80% power are set, the sample size is 7 animals/group (www.dssresearch.com/KnowledgeCenter/toolkitcalculators/samplesizecalculators.aspx). The sample size was slightly increased above this minimum number to have enough animals tested at the last follow‐up, considering animals' sacrifice (for histopathology) at the end of treatment.

### 2.3 Statistical Analysis

Descriptive statistics were generated for all variables. Normally distributed data were analyzed with parametric tests (*t‐test*) and non‐normally distributed data with non‐parametric tests (*Mann–Whitney U‐test*). Two‐sided tests were used. A *p* value < 0.05 was set as significant. All analyses were conducted in Graphpad environment (v8.0.2, La Jolla, CA, USA), apart from NET recordings. NET data were analyzed with QtraP (Institute of Neurology, Queen Square, London, UK), specifically designed to dialogue with the recording software QtraS (Institute of Neurology, Queen Square, London, UK).

### 2.4 Nerve Conduction Studies (NCS)

Recordings were performed on all animals/group following a standard published protocol that was demonstrated to be efficacious in detecting OIPN sensory changes at NCS [21]: this protocol encompasses two different sensory nerves from two different sites (caudal and digital nerve); notably, the digital nerve matches the dorsal sural nerve in humans, a distal sensory branch that was proven quite relevant in detecting OIPN sensory alterations at NCS [22]. Electromyography apparatus Myto2 (ABN Neuro, Firenze, Italy) and stainless‐steel needle electrodes were used (Subdermal EEG needle, Ambu, Ballerup, Denmark). All procedures were performed under deep isoflurane anesthesia while animal body temperature was monitored and kept constant at 37°C +/−0.5°C with a thermal pad, electronically connected to a thermal rectal probe (Harvard Apparatus, Holliston, US). For *distal caudal nerve sensory nerve action potential* (SNAP), the active recording electrode was placed at 5 cm from the tip of the tail and the reference recording electrode at 6 cm from it, the anode was placed at 1 cm from the tip of the tail and the cathode at 2 cm from it, the ground electrode was placed midway between the cathode and the active recording electrode. For *proximal caudal nerve* SNAP, the reference recording electrode was placed 1 cm from the base of the tail and the active recording electrode 2 cm from it, the cathode was placed at 5 cm from the base of the tail and the anode at 6 cm from it, the ground electrode was placed midway between the cathode and the active recording. For *digital nerve* SNAP, the reference recording electrode was placed in front of the patellar bone, the active recording electrode close to the ankle bone, the cathode was placed at the base of the fourth toe, and the anode was positioned at the tip of it; the ground electrode was placed in the sole. For *caudal nerve compound muscle action potential* (*CMAP*), the reference recording electrode was placed at 12 cm from the base of the tail and the active one at 11 cm from it, the ground electrode was placed at 8 cm from the base of the tail; for the distal stimulation site: the cathode was placed at 6 cm from the base of the tail and the anode at 5 cm from it, whereas for the proximal stimulation site, the cathode was placed at 2 cm from the base of the tail and the anode at 1 cm from it. Intensity, duration and frequency of stimulation were set up to obtain optimal results. For both sensory and motor recordings the peak‐to‐peak amplitude was considered. For sensory and motor nerve conduction velocity calculation, onset of SNAPs and CMAPs were considered. Averaging was applied carefully. For sensory recordings filters were kept between 20 Hz and 3KHz, for motor recordings between 20 Hz and 2 KHz. Sweep was kept at 0.5 msec. All procedures were carried out by an experienced operator, blind to the group allocation.

### 2.5 Recovery Cycle at Nerve Excitability Testing (NET)

NET was performed according to our previously published protocol for *caudal nerve*: the caudal nerve and its easily accessible course for the entire tail length was deemed ideal to favor a consistent montage among all animals [16, 18]. Rats were under deep isoflurane anesthesia, and the body temperature was kept constant at 37°C +/−0.5°C, ensuring to minimize all possible confounding factors due to an anesthesia‐related reduced body temperature. Briefly, this montage was used: for stimulation, disposable, non‐polarizable, Ag‐AgCl cup electrodes (Ambu, Ballerup, Denmark) were used with the conductive EEG paste (Ten20 Conductive EEG paste, Weaver and Company, Birmingham, AL). Depilatory cream (Veet, Slough, UK) was used for hair removal to allow positioning of the anode on the left hip. The cathode was set on the homolateral side of the tail, at 1.5 cm from its base. Skin was gently rinsed (Nuprep EEG & ECG Skin Prep Gel, Konan Medical, Irvine, CA) before placing cup electrodes, to guarantee appropriate cutaneous impedance. Stainless steel needle electrodes (Subdermal EEG needle, Ambu, Ballerup, Denmark) were used for ground and recording electrodes: active and reference recording electrodes were set at 6 and 2 cm distally to the cathode, respectively; the ground electrode was positioned midway between the cathode and active recording electrode.

As stimulator, an isolated linear bipolar constant current stimulant was used (maximal output +/−10 mA, DS4, Digitimer, Welwyn Garden City, UK); the Xcell3 Microelectrode Amplifier (FHC, Bowdoin, ME) was connected to the recording electrodes via a customized probe/adapter specifically designed by FHC for our needs; the National Instrument USB‐6221‐BNC Acquisition Device (National Instrument Italy, Assago, Italy) was used to connect all these instruments. For NET recordings the Qtrac software (Institute of Neurology, Queen Square, London, UK) and TROND protocol were used to acquire data on the recovery cycle. All procedures were carried out by an experienced operator, blind to the group allocation.

### 2.6 Behavioural Test—Dynamic Plantar Aesthesiometer

Mechanical allodynia was assessed using the Dynamic Plantar Aesthesiometer (Ugo Basile Biological Instruments, Varese, Italy). Each rat was placed in a clear plastic cage on a metal grid floor, and allowed to adapt to the testing environment for at least 15 min. Successively, a metal filament was applied to the plantar surface of the hind paw, exercising a linear increasing force ramp that reaches 50 g within 20 s. Mechanical threshold force was automatically registered three times for each paw and then calculated as the average of six consecutive values (expressed in grams). A 20 s cut‐off was fixed. All procedures were carried out by an experienced operator, blind to the group allocation.

### 2.7 IENF Density

Plantar glabrous skin biopsies were collected from the hind paws of three animals/group at both time points, immediately fixed in PLP (paraformaldehyde–lysine–sodium periodate) solution for 24 h at 4°C and cryoprotected at −20°C until use. A 20 μm sections were serially obtained with a cryostat. The sections were immunostained with rabbit polyclonal anti‐protein gene product 9.5 (UCHL1/PGP 9.5, Proteintech, Illinois, Rosemont, IL, USA) using a free‐floating protocol. The number of nerve fibers that cross the dermal‐epidermal junction was counted from three sections/animal under light microscopy, and the length of the epidermis was manually measured (Image J software, US National Institutes of Health) by an experienced operator blind to the group allocation. Finally, the IENF density (IENFD) was calculated as the total number of PGP 9.5 positive fibers/epidermal length (mm).

### 2.8 Nerve Morphology and Morphometry

Morphological and morphometric analyses were performed on distal caudal nerves, previously fixed by immersion in 3% glutaraldehyde and then post‐fixed in 1% OsO_4_. Once embedded in epoxy resin, 1.5 μm‐thin sections were obtained and stained with methylene blue. Images were acquired with a Nexcope Ne920 AUTO light microscope (TiEsseLab Srl, Milano, Italy) and all myelinated fibers evaluable were counted using a QWin automatic image analyzer (Leica Microsystems GmbH). The evaluation of fiber density was calculated on three animals/group both at the end of treatment and the follow‐up period.

### 2.9 Dorsal Root Ganglia (DRG) Morphometry and Morphology

Morphological and morphometric analysis of DRG was performed on sections from L4–L5 DRG, previously fixed by immersion in 4% paraformaldehyde and 2% glutaraldehyde and then post‐fixed in OsO4. Once embedded in epoxy resin, serial1.5 μm‐thin sections (spaced at 50‐μm intervals) were cut with a microtome and stained with methylene blue. Images were acquired with Nexcope Ne920 AUTO light microscope (TiEsseLab Srl, Milano, Italy).

Somatic, nuclear and nucleolar areas were manually measured using ImageJ software by a blind examiner (US National Institutes of Health). The evaluations were performed on three animals per group both at the end of the treatment and follow‐up period. At least 200 DRG neurons per animal were included in the analysis to reach statistical significance. Cells with a nucleolar area under 3 μm^2^ were excluded from the analysis. All procedures were carried out by an experienced operator, blind to the group allocation.

### 2.10 Spinal Cord Transient Receptor Potential Vanilloid Type‐1 (TRPV1), Calcitonin‐Related Peptide (CGRP) and Substance P (SP) Immunostainings

Paraffin‐embedded lumbar spinal cord segments were taken from 24 animals, divided into four groups: 6 at the end of the OHP treatment and 6 controls; 6 after a 4‐week follow‐up and 6 controls. Five‐μm‐thick spinal cord sections were deparaffinized with xylene, rehydrated and processed using the avidin‐biotin‐peroxidase complex (ABC) immunohistochemical technique. The endogenous peroxidase activity was blocked by 0.1% phenylhydrazine (Cat# 101326606, Sigma Aldrich, St Louis, MO, USA) in phosphate‐buffered saline (PBS) containing 0.2% Triton X‐100 (PBS/T). Antigen retrieval was then achieved by heating at 90°C for 20 min in 10 mM citrate buffer (pH 6.0), followed by gradual cooling for 20 min. The sections were then incubated with 20% normal goat serum (Cat# S‐1000, Vector, Burlingame, CA, USA). Rabbit polyclonal antibodies against TRPV1 (Cat# ab10296, AbCam, Cambridge, UK), diluted 1:600, and Calcitonin gene‐related peptide (CGRP) (#CA1134, Enzo Life Sciences), diluted 1:1000, and a rat polyclonal antibody against Substance P (SP) (#sc‐21 715, SantaCruz Biotechnology, CA, USA), diluted 1:800, were used as the primary antibodies. Biotin‐conjugated goat anti‐rabbit (BA‐1000, Vector, Burlingame, CA, USA) and anti‐rat antisera (BA‐6000, Vector, Burlingame, CA, USA), whose respective dilutions were 1:400 and 1:600, were used as the secondary antiserum. The reaction product was revealed by the ABC (Cat#G011‐61, BioSpa Div., Milan, Italy), diluted 1:250, followed by incubation with a solution of 0.1 M PB, pH 7.3, containing 0.05% 3,3′‐diaminobenzidine (Sigma Aldrich, St Louis, MO, USA), 0.04% nickel ammonium sulphate and 0.01% hydrogen peroxide. All the antisera and the ABC were diluted in PBS/T. Incubation with primary antibodies was carried out for 24 h at 4°C. Incubations with secondary antiserum and ABC lasted 60 and 40 min, respectively, and were performed at room temperature. Negative control preparations were obtained by incubating tissue sections in parallel with PBS/T alone or by omitting the primary antibody. Slides were observed using an Olympus B×61 microscope, and digital images were captured with a Leica DFC450C camera.

### 2.11 TRPV1 Image Densitometry

For the quantitative evaluation of the TRPV1 immunohistochemical labeling in the dorsal horn of the spinal cord, 12 representative 10× magnification microscopic fields from each group were blindly analyzed with ImageJ (http://rsb.info.nih.gov/ij/). Mean gray values from negative controls were subtracted from the gray values of the immunostained sections to exclude background staining. Graphs were obtained by calculating the ratio of OHP‐treated and OHP follow‐up values to control values.

## 3 Results

### 3.1 Neurophysiological Recordings

Descriptive statistics at *base line* for NCS are reported in Table 1, as well as Mann–Whitney U‐test: no difference was observed between the 2 groups for all variables. *End of treatment* descriptive statistics for NCS are reported in Table 2, as well as Mann–Whitney U‐test results: distal caudal and digital SNAP amplitude were significantly reduced in OHP group compared to controls (*p* value 0.008 and 0.001, respectively). Recovery cycle NET findings and statistical test results at this same time point are shown in Table 3: there was a difference between the 2 groups only for recovery cycle parameters with regard to superexcitability (%), superexcitability at 7 and 5 msec (*p* value respectively: < 0.005, < 0.001, 0.02). At *1 week follow‐up* after chemotherapy completion, recovery cycle NET findings and statistical test results are shown in Table 4: no difference was still detectable between the 2 groups and, therefore, no signs of axonal hyperexcitability were observed. At *6‐week follow‐*up after chemotherapy completion descriptive statistics for NCS are reported in Table 5, as well as Mann–Whitney U‐test results: no differences were seen between the groups. NET findings and statistical test results are shown in Table 6: no difference was demonstrated between the 2 groups.

**TABLE 1:** Nerve conduction studies at baseline. Descriptive statistics and Mann–Whitney U‐test results are shown.

VARIABLE	Median	Q1	Q3	*p*
DC_AMP in CTRL group	53.95	47.10	58.30	0.165
DC_AMP in OHP group	51.15	46.00	52.90	
DC_VEL in CTRL group	27.80	26.80	28.80	0.579
DC_VEL in OHP group	27.15	26.10	28.60	
PC_AMP in CTRL group	128.35	116.50	140.30	0.190
PC_AMP in OHP group	143.50	123.50	152.90	
PC_VEL in CTRL group	35.30	33.30	36.10	0.315
PC_VEL in OHP group	36.20	34.90	37.50	
D_AMP in CTRL group	58.00	52.30	62.20	0.353
D_AMP in OHP group	55.40	52.00	58.30	
D_VEL in CTRL group	35.20	33.30	36.30	0.529
D_VEL in OHP group	34.15	33.70	35.40	
M_AMP in CTRL group	5.65	4.80	6.00	0.089
M_AMP in OHP group	7.45	6.10	8.00	
M_VEL in CTRL group	37.00	33.30	37.70	0.796
M_VEL in OHP group	35.30	33.90	38.50	

*Note:* DC_AMP: distal caudal nerve sensory nerve action potential (SNAP) amplitude (μV); DC_VEL: distal caudal nerve sensory conduction velocity (m/s); D_AMP: digital nerve SNAP amplitude (μV); D_VEL: digital nerve sensory conduction velocity (m/s); M_AMP: caudal nerve compound muscle action potential (CMAP) amplitude (mV); M_VEL: caudal nerve motor conduction velocity; PC_AMP: proximal caudal nerve SNAP amplitude (μV); PC_VEL: proximal caudal nerve sensory conduction velocity (m/s).

**TABLE 2:** Nerve conduction studies at the end of treatment. Descriptive statistics and Mann–Whitney U‐test results are shown.

VARIABLE	Median	Q1	Q3	*p*
DC_AMP in CTRL group	58.30	51.40	63.40	**0.008**
DC_AMP in OHP group	44.50	42.00	53.80	
DC_VEL in CTRL group	30.60	29.70	33.30	0.113
DC_VEL in OHP group	29.25	27.80	30.00	
PC_AMP in CTRL group	132.70	129.00	136.40	0.211
PC_AMP in OHP group	125.90	117.30	132.60	
PC_VEL in CTRL group	38.50	35.30	40.00	0.497
PC_VEL in OHP group	37.50	36.60	38.50	
D_AMP in CTRL group	80.70	65.50	85.00	**0.001**
D_AMP in OHP group	55.95	48.10	62.10	
D_VEL in CTRL group	37.80	36.80	40.00	0.720
D_VEL in OHP group	37.55	36.90	39.40	
M_AMP in CTRL group	6.30	5.50	6.80	0.968
M_AMP in OHP group	5.70	4.30	7.50	
M_VEL in CTRL group	40.00	39.20	41.70	0.315
M_VEL in OHP group	38.45	37.00	40.80	

*Note:* DC_AMP: distal caudal nerve sensory nerve action potential (SNAP) amplitude (μV); DC_VEL: distal caudal nerve sensory conduction velocity (m/s); D_AMP: digital nerve SNAP amplitude (μV); D_VEL: digital nerve sensory conduction velocity (m/s); M_AMP: caudal nerve compound muscle action potential (CMAP) amplitude (mV); M_VEL: caudal nerve motor conduction velocity; PC_AMP: proximal caudal nerve SNAP amplitude (μV); PC_VEL: proximal caudal nerve sensory conduction velocity (m/s). Significant *p*‐values are shown in bold.

**TABLE 3:** Recovery Cycle parameters at NET at end of treatment. Descriptive statistics and Mann–Whitney U‐test results are shown.

VARIABLE	CTRL GROUP [median, (Q1, Q3)]	OHP GROUP [median, (Q1, Q3)]	Mann–Whitney U‐test
Relative Refractory Period (msec)	0.386 (0.365, 0.47)	0.486 (0.407, 0.569)	***p* = 0.04**
Superexcitability (%)	−13.9 (−15.2, −13)	−6.18 (−9.16, −4.34)	***p* < 0.005**
Subexcitability (%)	5.07 (3.12, 5.37)	5.51 (4.12, 9.8)	*p* = 0.25
Superexcitability at 5 msec	−13.9 (−16.4, −12.5)	−3.41 (−10.4, −1.04)	***p* = 0.02**
Superexcitability at 7 msec	−11.9 (−13.2, −8.34)	−4.32 (−5.84, −3.02)	***p* < 0.001**
Refractoriness at 2 msec	12.3 (9.01, 30.7)	34.9 (18.9, 48)	*p* = 0.07
Refractoriness at 2.5 msec	−2.17 (−4.57, 8.25)	8.96 (−0.516, 15)	*p* = 0.07

*Note:* Significant *p*‐values are shown in bold.

**TABLE 4:** Recovery Cycle parameters at NET 1 week after treatment. Descriptive statistics and Mann–Whitney U‐test results are shown.

VARIABLE	CTRL GROUP [median, (Q1, Q3)]	OHP GROUP [median, (Q1, Q3)]	Mann–Whitney U‐test
Relative Refractory Period (msec)	0.295 (0.277, 0.387)	0.383 (0.303, 0.485)	*p* = 0.79
Superexcitability (%)	−11.7 (−13.9, −11.6)	−9.62 (−10.4, −7.48)	*p* = 0.33
Subexcitability (%)	2.08 (1.37, 2.65)	5.17 (3.83, 9.38)	*p* = 0.18
Superexcitability at 7 msec	−8.32 (−9.86, −4.32)	−7.04 (−8.53, −4.75)	*p* = 0.79
Superexcitability at 5 msec	−11.1 (−11.4, −4.96)	−8.81 (−11.1, −6.24)	*p* = 0.93
Refractoriness at 2 msec	−2.15 (−3.4, 12.3)	12.9 (−2.5, 17.3)	*p* = 0.79
Refractoriness at 2.5 msec	−8.99 (−10.6, −2.26)	−2.32 (−7.78, 5.4)	*p* = 0.43

**TABLE 5:** Nerve conduction studies at 6 weeks of follow‐up after treatment. Descriptive statistics and Mann–Whitney U‐test results are shown.

VARIABLE	Median	Q1	Q3	*p*
DC_AMP in CTRL group	95.80	65.90	98.40	0.177
DC_AMP in OHP group	65.25	60.60	66.60	
DC_VEL in CTRL group	33.00	32.30	34.10	0.329
DC_VEL in OHP group	32.15	31.30	33.00	
PC_AMP in CTRL group	154.20	148.00	161.70	0.429
PC_AMP in OHP group	149.85	144.90	153.80	
PC_VEL in CTRL group	42.90	42.30	43.50	0.662
PC_VEL in OHP group	42.60	41.70	42.90	
D_AMP in CTRL group	87.10	78.60	94.20	0.662
D_AMP in OHP group	87.75	80.50	95.80	
D_VEL in CTRL group	40.30	40.30	40.80	0.126
D_VEL in OHP group	41.70	41.20	42.00	
M_AMP in CTRL group	5.00	3.90	5.40	0.429
M_AMP in OHP group	5.75	4.80	7.80	
M_VEL in CTRL group	41.70	40.00	42.60	0.792
M_VEL in OHP group	41.25	40.80	42.60	

*Note:* DC_AMP: distal caudal nerve sensory nerve action potential (SNAP) amplitude (μV); DC_VEL: distal caudal nerve sensory conduction velocity (m/s); D_AMP: digital nerve SNAP amplitude (μV); D_VEL: digital nerve sensory conduction velocity (m/s); M_AMP: caudal nerve compound muscle action potential (CMAP) amplitude (mV); M_VEL: caudal nerve motor conduction velocity; PC_AMP: proximal caudal nerve SNAP amplitude (μV); PC_VEL: proximal caudal nerve sensory conduction velocity (m/s).

**TABLE 6:** Recovery Cycle at NET 6 weeks after treatment completion. Descriptive statistics and Mann–Whitney U‐test results are shown.

VARIABLE	CTRL GROUP [median, (Q1, Q3)]	OHP GROUP [median, (Q1, Q3)]	Mann–Whitney U‐test
Relative Refractory Period (msec)	0.277 (0.272, 0.318)	0.367 (0.332, 0.389)	*p* = 0.34
Superexcitability (%)	13.1 (−14.9, −11.9)	−13.9 (−14.6, −11.8)	*p* = 1
Subexcitability (%)	1.27 (1.17, 2.74)	1.79 (1.32, 2.23)	*p* = 0.88
Superexcitability at 7 msec	−10.2 (−10.9, −9.5 *n* = 4)	−12.3 (−12.8, −10.6)	*p* = 0.34
Superexcitability at 5 msec	−11.6 (−12.5, −11.3)	−13.8 (−14.8, −12.1)	*p* = 0.68
Refractoriness at 2 msec	−3.17 (−4.03, 0.661)	6.44 (2.95, 10.2)	*p* = 0.48
Refractoriness at 2.5 msec	−9.61 (−11.9, −5.91)	−3.35 (−7.42, −0.884)	*p* = 0.68

### 3.2 Mechanical Allodynia

**FIGURE 1:**
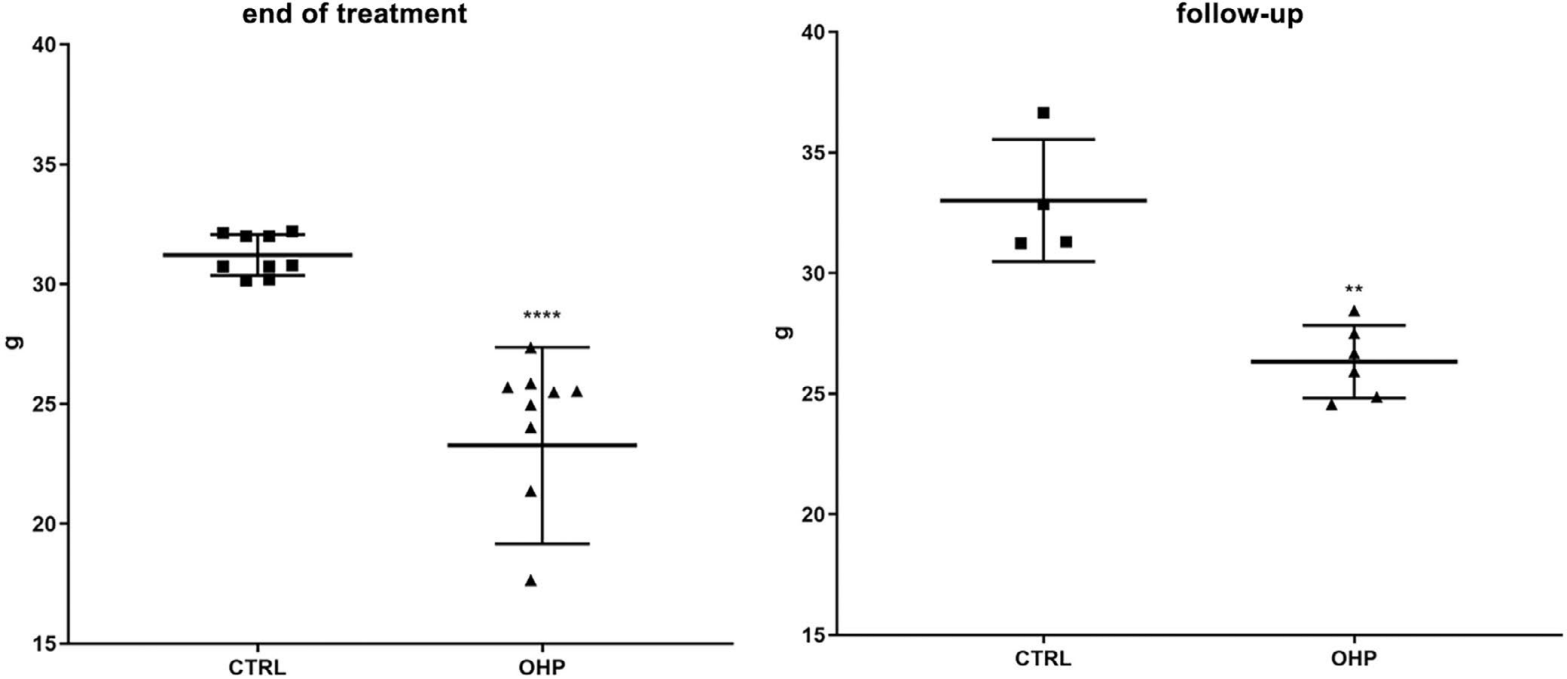
Dynamic Plantar Aesthesiometer test. OHP group showed a significant reduction in mechanical threshold (expressed in grams) both at the end of OHP treatment and after the 6‐week follow‐up, despite a certain degree of amelioration for the latter time point (****p < 0.0001 end of treatment, left side, **p < 0.01 end of follow‐up, right side vs. CTRL, Mann–Whitney U‐test).

A significant reduction in mechanical threshold was observed in OHP‐treated animals at the end of treatment (Figure 1, left side, *p* < 0.0001) which persisted to the end of the follow‐up period although with less severity (Figure 1, right side, *p* < 0.01).

### 3.3 IENF Density

**FIGURE 2:**
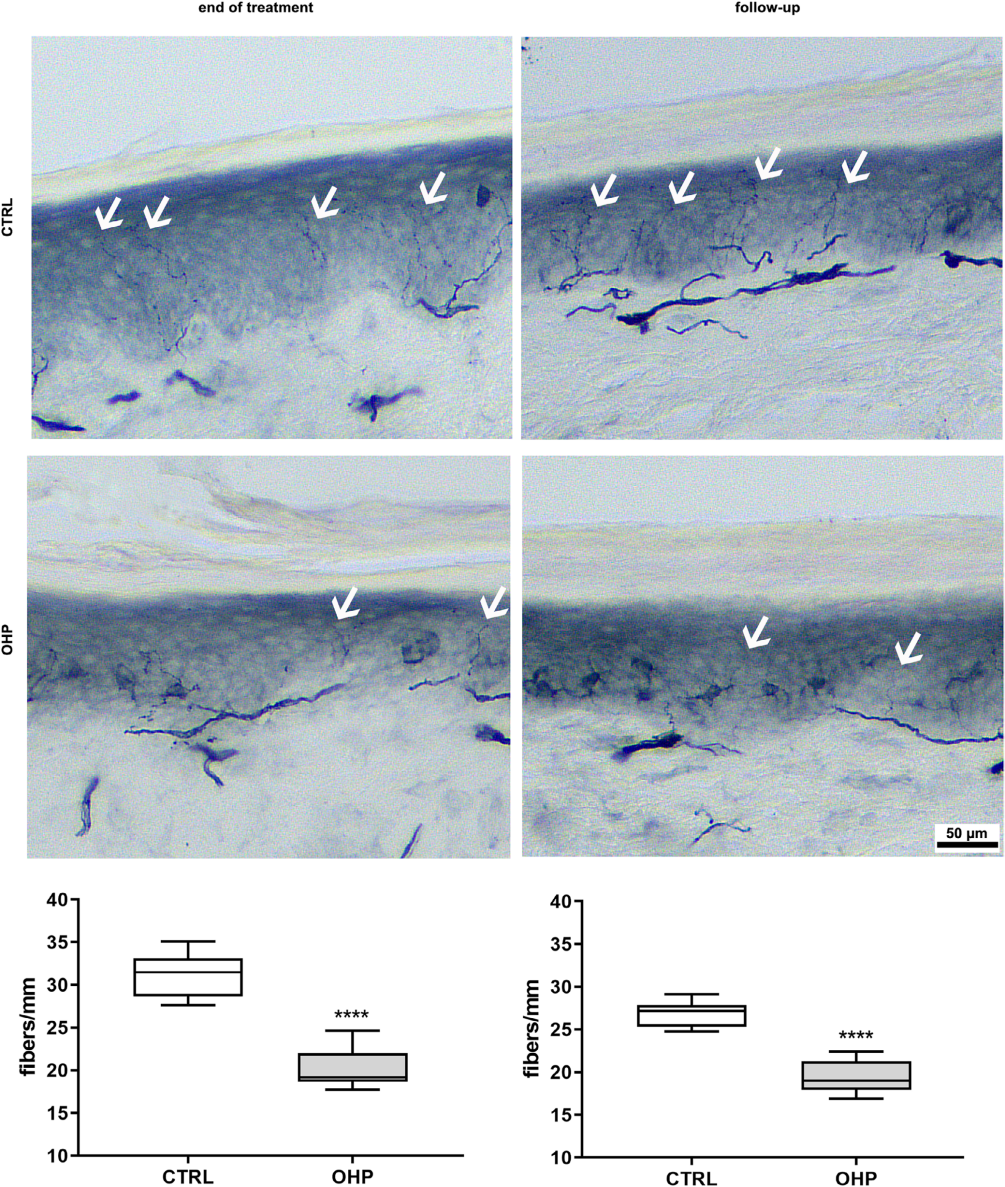
Intraepidermal nerve fiber (IENF) density. In the upper part of the image, representative photos of CTRL and OHP animals are shown both at end of treatment and the 6‐week follow‐up: White arrows point out the intraepidermal nerve fibers. In the bottom part, graphs show a significant reduction in IENF density both at the end of OHP treatment and after follow‐up period (****p < 0.0001 vs. CTRL, Mann–Whitney U‐test).

A severe reduction in IENF density was detected both after the 4‐week treatment and the follow‐up period (Figure 2, *p* < 0.0001).

### 3.4 Nerve Morphology and Morphometry

**FIGURE 3:**
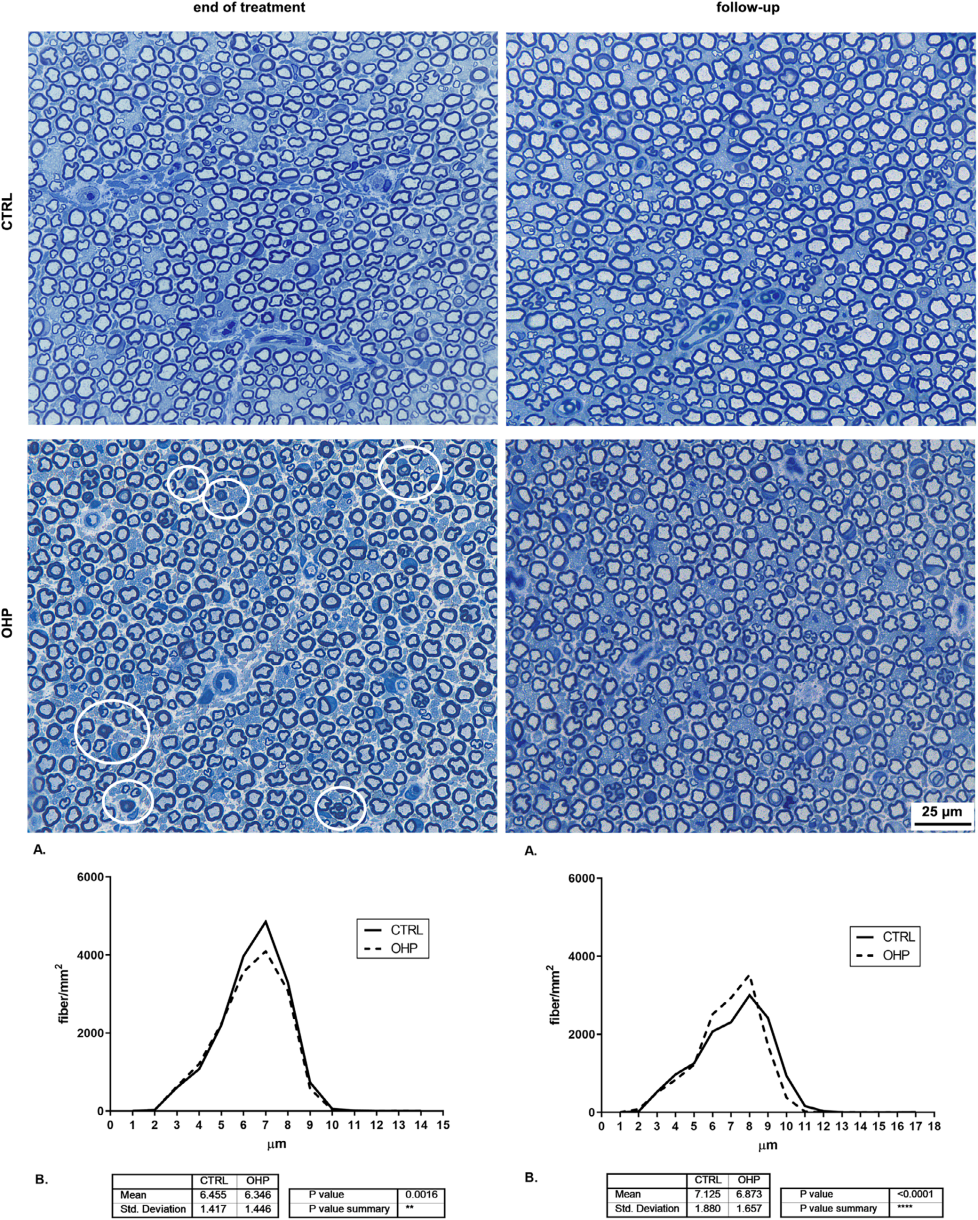
Nerve morphology and morphometry of the distal caudal nerve. In the upper part of the image, representative photos of CTRL and OHP animals are shown both at end of treatment and the 6‐week follow‐up: White circles point out representative degenerated fibers. In the bottom part, Panels A represent fiber density distribution for both CTRL and OHP groups. Panels B, instead, represents the mean fiber diameter for both groups and the results of the *t*‐test for this value (**p < 0.01 end of treatment, left side, ****p < 0.0001 end of follow‐up vs. CTRL, right side, *t*‐test).

After 4 weeks of OHP treatment, histopathological examinations of the distal portion of caudal nerves showed mild alterations, which appeared even less evident after 6 weeks from the end of the treatment (Figure 3). Morphometric analysis of the distribution of myelinated fibers showed a reduction in the number of medium‐caliber fibers after OHP treatment (Figure 3A, left side) whereas a mild loss of large‐caliber nerve fibers was observed in OHP‐treated animals compared to control at follow‐up (Figure 3A, right side). In addition, a significant reduction in the mean fiber diameters was detected both after OHP treatment (*p* < 0.01; Figure 3B, left side) and the follow‐up period (*p* < 0.0001; Figure 3B, right side).

### 3.5 DRG Morphology and Morphometry Results

**FIGURE 4:**
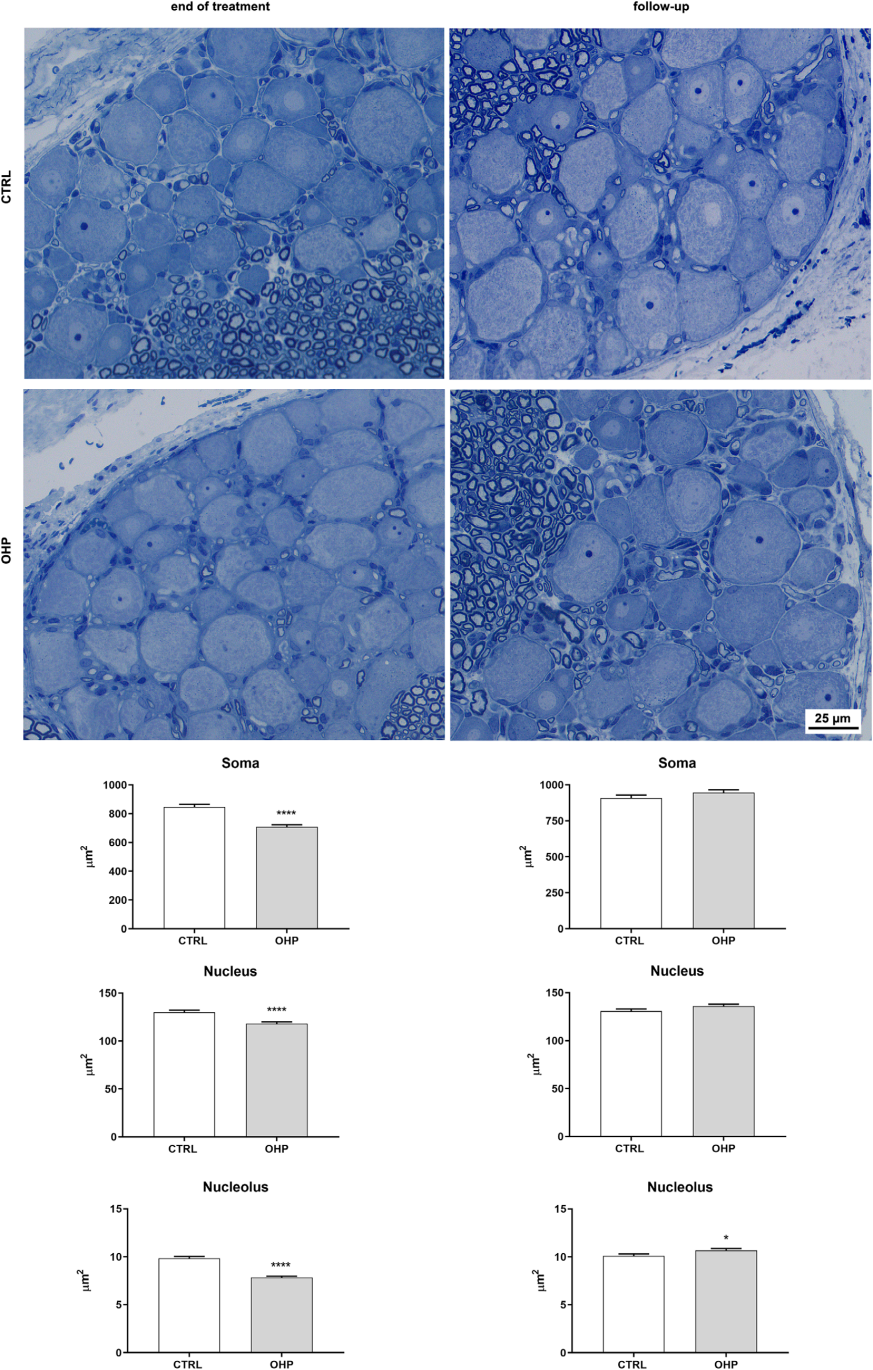
Dorsal root ganglia (DRG) morphology and morphometry. In the upper part of the image, representative photos of CTRL and OHP animals are shown both at the end of treatment and the 6‐week follow‐up. In the bottom part, graphs are shown representative of the statistical significance (****p < 0.0001 end of treatment, left side, *p < 0.01 end of follow‐up, right side vs. CTRL, *t*‐test).

Morphology and morphometry analyses were performed to study the structural changes in DRG sensory neurons. Representative images of DRG are shown (Figure 4). After 4 weeks of treatment, DRG neurons from the OHP‐treated group showed a statistically significant decrease in mean area for somatic (*p* < 0.0001), nuclear (*p* < 0.0001) and nucleolar (*p* < 0.0001) areas compared to the control group (Figure 4, left side). After 6 weeks of follow‐up a recovery in both somatic and nuclear areas was observed. Only the nucleolar mean area was slightly increased (*p* = 0.0475) in the OHP‐treated group compared to the control group at the end of follow‐up (Figure 4, right side).

### 3.6 Spinal Cord TRPV1 and Neuropeptide Immunostainings

**FIGURE 5:**
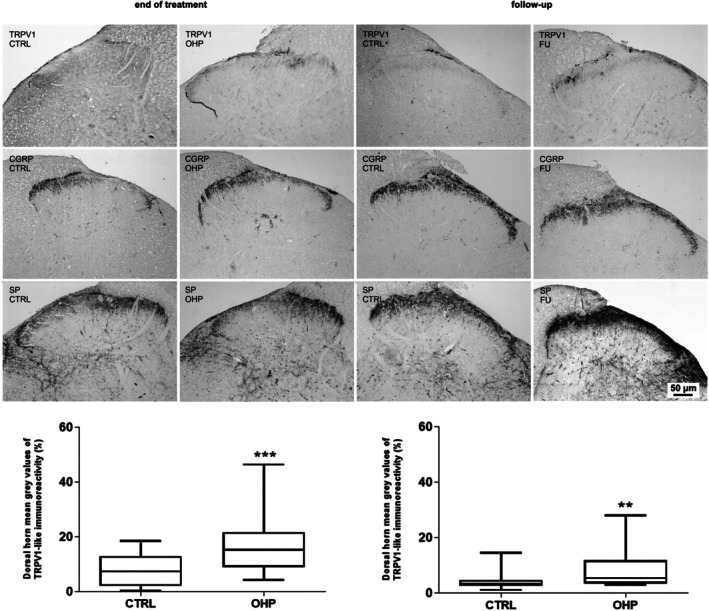
TRPV1‐, CGRP‐, and SP‐like immunoreactivity in the dorsal horn of the spinal cord. In the upper part of the image, representative photos of CTRL and OHP animals are shown both at the end of treatment and the 6‐week follow‐up. In the bottom part, graphs are shown representative of the statistical significance (***p < 0.001 end of treatment, left side, **p < 0.01 end of follow‐up, right side vs. CTRL, *t*‐test).

The immunoreactivity to TRPV1 and the neuropeptides CGRP and SP was found in the dorsal horn (Figure 5). TRPV1‐immunostained structures were represented by nerve fibers in Lissauer's tract and lamina I and by a lighter, diffuse immunostaining in inner lamina II (Figure 5A, first row). CGRP‐ and SP‐like immunoreactivity displayed a wider distribution pattern and labeled nerve fibers and terminals in Lissauer's tract, laminae I–III, and lamina V (Figure 5A, second and third row, respectively). Densitometric analysis of TRPV1 immunolabeling in the dorsal horn at the end of treatment showed an increased density of TRPV1 staining in OHP animals (Figure 5B). The unpaired *t*‐test confirmed a statistically significant increase of TRPV1‐LI after the end of treatment versus the control animals (*p* = 0.0005) (Figure 5, left side). This difference was maintained on samples harvested at follow‐up (Figure 5) and confirmed by the *t*‐test that showed a statistically significant increase of TRPV1‐LI after the six‐week follow‐up (*p* = 0.0045) (Figure 5, right side).

## 4 Discussion

OIPN full clinical spectrum comprises painful manifestations that can arise both from its acute and chronic forms. Our study was designed to fully characterize them both: to this end we relied on a multimodal approach to detect all possible aspects of OIPN in a robust rat model. Our study design, in fact, was tailored following the detailed methodological approach suggested by Bruna et al. [23] that highlighted the need to incorporate neuropathology, neurophysiology and behavioral tests to appropriately characterise CIPN in rodent models. NCS and behavioral tests at the end of treatment as well as morphology/morphometry of both caudal nerves and DRG were able to demonstrate that our schedule efficiently induced the expected axonal damage. NET parameters of the recovery cycle at the end of 4‐week treatment were in line with our previously published data and demonstrated that acute OIPN (i.e., a transient functional channelopathy) had ensued [16, 18]. Remarkably, alterations at recovery cycle were transient and didn't mirror the ongoing axonal damage: in fact, 1 week after treatment NET parameters were back to normal in the OHP‐treated group. We then prolonged the observation time of our cohort up to 6 weeks after treatment: once again NET showed no signs of a functional channelopathy being persistent, whereas chronic OIPN was still detectable, despite it encountering an amelioration, mostly for what regards the involvement of large myelinated fibers that can be studied via NCS. Notably, damage to small unmyelinated fibers remained significant at both time points as demonstrated by IENFD assessment and reinforced by behavioral testing; thus, our model is well‐suited to shed light into the full spectrum of acute and chronic painful OIPN manifestations. To provide even stronger evidence that IENFD and behavioral test results indicated a persistent neuropathic pain, we expanded our analyses to include spinal cord TRPV1, CGRP, and SP immunostainings. It is important to note that reduced IENFD does not necessarily equal chronic neuropathic pain [24, 25], nor does behavioral testing directly measure neuropathic pain, as it potentially primarily reflects a nocifensive behaviour in animals [26, 27]. TRPV1 detection is consistent with the scope of our study, as it is a non‐selective cation channel with a pivotal role in sensory neurons both in transmission and modulation of nociceptive signals. TRPV1 is activated and/or sensitized in conditions of nerve injury [28, 29, 30]. CGRP, in contrast, is a specific marker for neuropeptidergic neurons which are primarily associated with unmyelinated fibers (C fibers) and polymodal nociceptors [31]. Most neurons expressing SP also fall into this category. Moreover, both CGRP and SP are expressed by medium‐sized neurons connected with thinly myelinated (A delta) axons, which predominantly function as high‐threshold mechanoreceptors [31, 32]. From a functional standpoint, these three elements are interconnected: TRPV1 activation leads to neuronal depolarisation, the onset of pain, and subsequent release of CGRP and SP. These neuropeptides act on the effector cell receptors enhancing nociceptor sensitization [31, 32]. Our immunohistochemistry data in the OHP group showed a more intense TRPV1 immunolabeling in Lissauer's tract, lamina I, and inner lamina II both at the end of treatment and at the end of follow‐up. This pattern was mirrored by a more intense CGRP‐ and SP‐like immunoreactivity, which displayed a wider distribution and labeled nerve fibers and terminals in Lissauer's tract, laminae I‐III, and lamina V. Taken together with IENFD results and behavioural test data, these findings robustly indicate that chronic neuropathic pain persisted in the OHP group both at the end of chemotherapy and after 6 weeks of follow‐up. Our results are in line with previously published studies, where similar immunolabeling patterns were used to characterize painful features in another CIPN rodent model induced by bortezomib, whose hallmark is a relevant neuropathic pain component [32, 33].

Our findings suggest that painful features due to acute OIPN can be considered as a separate entity with respect to a chronic condition linked to neuropathic pain: the former is a transient dysfunction in ion channels, whereas the latter is linked to actual axonal damage in small unmyelinated fibers. This might be of interest since over the last decade data suggested that these acute functional alterations are nevertheless not irrelevant since, despite being transient, they might be linked to a more severe chronic OIPN [9, 34], and targeting the acute OIPN might have a beneficial effect on chronic OIPN too [18, 19].

This piece of information could be considered both at bench and bed side. At bed side, in fact, a proper assessment of acute OIPN is possible via NET too. Of course, NET is quite complex, but, at least in a research setting, it could be an option to be used as a translational tool to detect acute OIPN not only at bench side but also at bed side, since it was originally developed at a clinical level [35]. However, it should be recognized that full NET recording requires 10–15 min per patient, causes some discomfort, and demands skilled personnel and specialized equipment (i.e., specific hardware and software that are not part of the standard equipment of clinical neurophysiology units). Nevertheless, a tentative diagnostic approach can be proposed to characterize both acute and chronic OIPN: a detailed history supported by the specific Oxaliplatin‐questionnaire [34, 36] for painful symptoms related to transient axonal hyperexcitability not linked to axonal damage, combined with a simple and validated scale, as the neuropathic pain diagnostic questionnaire (DN4), to detect chronic neuropathic pain, whose specificity and sensitivity both exceed 90% [37].

Raising the attention to the possibility of discriminating between painful manifestations related to acute and chronic OIPN might be useful since not all health‐care professionals in charge of cancer patients have a specific training in the pain field and they might eventually misinterpret acute OIPN painful manifestations leading to overtreatment, thus applying the standard of care for chronic neuropathic pain even if only acute OIPN had ensued [38]. Furthermore, it is something to be considered in clinical trials: to carefully weigh which efficacy endpoint to select to discriminate acute and chronic OIPN, using, for example, the two scales already mentioned. Last, but not least, given that acute OIPN, if more pronounced, might be a red flag that patients could develop a more relevant chronic OIPN [34], its assessment might guide patients' surveillance during chemotherapy.

Overall, our data suggest that a well‐rounded characterization of all the phenomena under the broader OIPN full spectrum can be taken into consideration in clinical and preclinical studies, since acute and chronic OIPN contribute in different ways to painful symptoms and, eventually, to actual axonal damage.

## Conflicts of Interest

The authors declare no conflicts of interest.

## Data Availability

The data that support the findings of this study are available from the corresponding author upon reasonable request.
